# KBG syndrome: report and follow-up on three unrelated patients observed at different ages

**DOI:** 10.1186/s13052-025-01884-1

**Published:** 2025-02-21

**Authors:** Gregorio Serra, Pierandrea Elefante, Ylenia Gazzitano, Luigi Memo, Valeria Mineo, Carla Morando, Rosaria Nardello, Ettore Piro, Laura Travan, Giovanni Corsello

**Affiliations:** 1 Department of Health Promotion, Mother and Child Care, Internal Medicine and Medical Specialties “Giuseppe D’Alessandro”, University of Palermo, Palermo, Italy; 2 IRCCS Institute for Maternal and Child Health “Burlo Garofolo”, Trieste, Italy; 3 Department of Pediatrics, “San Bortolo” Hospital, Vicenza, Italy

**Keywords:** *ANKRD11* gene, array-CGH, Next generation sequencing, Sanger sequencing, 16q24.3 deletion

## Abstract

**Background:**

KBG syndrome (MIM #148050) is a rare genetic disease, showing an autosomal dominant pattern of inheritance. It was first described by Herrmann et al. in 1975 in three affected families, whose initial letters gave origin to the acronym. A peculiar *facies* including triangular face, synophrys, macrodontia of the upper central incisors, as well as short stature, skeletal defects and neurodevelopmental disorders (developmental delay, intellectual disability, epilepsy) are the main features of the syndrome. Mutations of the ankirin repeat domain 11 gene (*ANKRD11)*, which harbors at chromosome 16q24.3, have been associated to the syndrome. The encoded protein inhibits ligand-dependent activation of transcription. Due to the growing number of detected *ANKRD11* variants associated to phenotypes with various degree of severity, the precise definition of the clinical and genomic profiles of patients is important, also in the perspective of a better understanding of the molecular bases of the disease, genotype-phenotype correlation, and management of affected subjects.

**Cases presentation:**

We report on three unrelated patients, observed in as many different Italian (Sicily, Veneto and Friuli-Venezia-Giulia regions) Pediatric Neurology and Medical Genetics outpatient services, showing variously present typical dysmorphic features (e.g., triangular face, macrodontia of upper incisors, brachydactyly), growth retardation and impaired neurodevelopmental profiles (i.e. developmental delay, EEG abnormalities/epilepsy) compatible with KBG syndrome diagnosis. In Patient 1, next generation sequencing analysis of a panel of genes involved in developmental delay and autism spectrum disorders detected two mutations, a pathogenic heterozygous frameshift variant of the *ANKRD11* gene (already described in the literature), and a heterozygous missense one in *EHMT1* (previously reported as well, and associated with Kleefstra syndrome); in Patient 2, array comparative genomic hybridization (a-CGH) analysis identified a 634 Kb 16q24.3-24.3 deletion involving several genes (*CDT1*,* APRT*,* GALNS*,* TRAPPC2L*,* ACSF3*,* CDH15*), besides *ANKRD11*, some of which are related with developmental disorders. Finally in Patient 3, Sanger sequencing of the *ANKRD11* gene, performed due to the specific diagnostic suspicion raised for precocious teething observed at age 3 months, evidenced an intragenic deletion allowing thus an early diagnosis of disease.

**Conclusions:**

We underline similarities and differences among our patients, and their specific genetic and clinical features, in addition to the variable diagnostic tests used for the diagnosis, reached at different developmental age, i.e. infancy, childhood and adolescence. Pediatricians must be aware of KBG syndrome and should be able, as well, to raise the diagnostic suspicion, especially in the presence of peculiar dysmorphic features, short stature, developmental delay, intellectual disability and epilepsy. Prompt diagnosis may allow to better address any associated emerging neuropsychological and behavioral issues improving the quality of life of the patient and the whole family.

## Background

KBG syndrome (MIM #148050) is a rare genetic disease, showing an autosomal dominant pattern of inheritance. Over 200 cases have been identified to date, with novel diagnoses becoming increasingly more frequent according with the growing clinical availability of the new genomic analysis tools [1]. The syndrome is characterized by peculiar craniofacial features including triangular face, synophrys, macrodontia of the upper central incisors, in addition to short stature, skeletal defects, and neurological anomalies which enclose developmental delay, intellectual disability and epilepsy [2]. It was first described by Herrmann et al. in 1975 in three unrelated families, whose initial letters gave origin to the acronym [3]. Mutations of the ankirin repeat domain 11 gene (*ANKRD11)*, encoding a 298 kDa protein of 2663 amino acids and which harbors at chromosome 16q24.3, have been associated to the disease [4]. The severity of the clinical phenotype does not appear related with the type of variant, and no clear genotype–phenotype correlations have been established to date [2]. However, either heterozygous mutations in *ANKRD11* or deletions of 16q24 including such gene may be found in KBG patients, with both mechanisms likely leading to similar phenotypes [5, 6]. Due to the growing number of detected *ANKRD11* variants associated to phenotypes with various degree of severity, the precise definition of the clinical and genomic profiles of the syndrome is of the utmost importance. Such deeper characterization is useful in the perspective of both a better understanding of the underlying pathogenetic and molecular mechanisms of the disease [7], and management of affected subjects.

We report on three unrelated patients, observed in different Italian (Sicily, Veneto, and Friuli-Venezia-Giulia regions) Pediatric Neurology and Medical Genetics outpatient services, showing dysmorphic features, growth retardation and impaired neurodevelopmental profile (i.e. developmental delay, EEG abnormalities/epilepsy) compatible with KBG syndrome diagnosis, confirmed by different molecular tests, i.e. Next Generation Sequencing (NGS), array comparative genomic hybridization (a-CGH) and Sanger sequencing of the *ANKRD11* gene. We underline similarities and differences among our patients, for both the specific genetic and clinical characteristics, and the variable diagnostic paths which allowed the identification of the disease at different developmental ages.

## Cases presentation

### Patient 1

**Fig. 1a/b/c:**
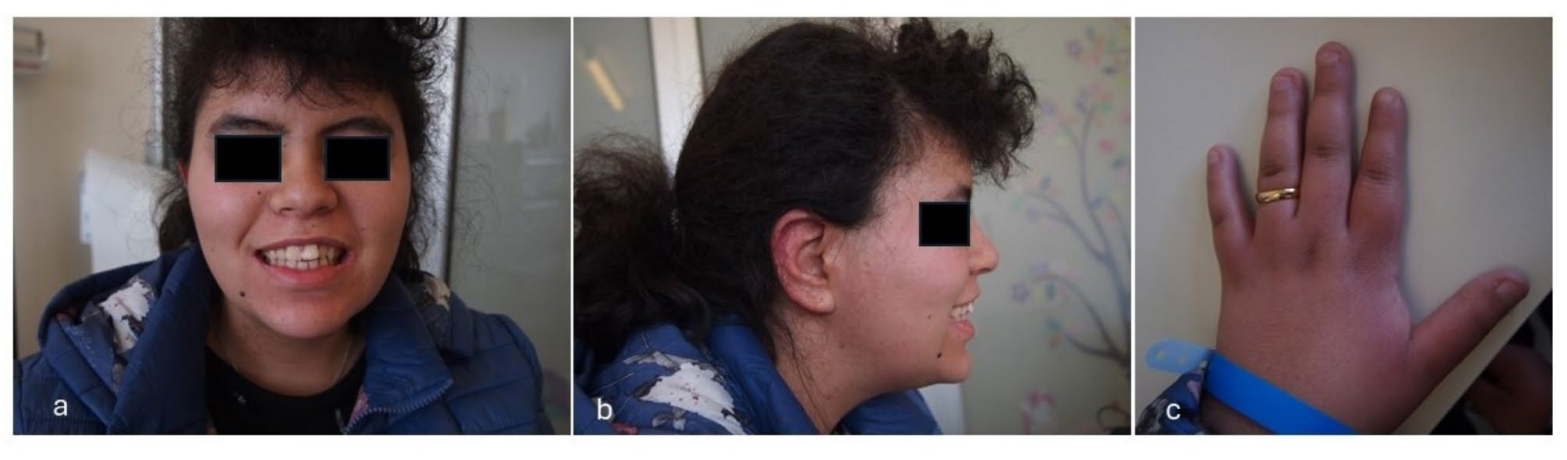
Patient 1. **a** Squared face due to chubby cheeks and enlarged chin, curly hair, thick eyebrows and synophrys, hypertelorism, mild epicanthus, wide nasal bridge and bulbous tip, hypoplastic and short philtrum, macrodontia of permanent upper central incisors with dental crowding, and thick lower lip. **b** Wide and prominent ears with hypoplastic antihelix. **c** brachydactyly of the 1st, 2nd and 5th finger, with clinodactyly of the latter

The proband is an eighteen-year-old girl. She was born in Brazil and arrived in Italy when she was one year and six months old, following her adoption. Her family and perinatal history were fragmentary and poorly known: she had a paternal uncle affected by epilepsy (whose further clinical data is not available) and a healthy sister, coming with her to Italy after the adoption by another family. At our first examination, anthropometric measurements were as follows: weight 74 Kg (95th centile, + 1.65 standard deviations, SD), height 154 cm (6th centile, -1.58 SD), occipitofrontal circumference (OFC) 56 cm (63rd centile, + 0.3 SD), according to World Health Organization growth charts [8]. She showed squared face due to chubby cheeks and enlarged chin, curly hair, wide and prominent ears with hypoplastic antihelix, thick eyebrows and synophrys, hypertelorism, mild epicanthus, wide nasal bridge and bulbous tip, hypoplastic and short philtrum, macrodontia of permanent upper central incisors with dental crowding, and thick lower lip. In addition, bilateral brachydactyly of the 1st, 2nd and 5th fingers, with clinodactyly of the latter, was noted (Fig. 1a/b/c).

She acquired independent walking at 20 months, while a language impairment along with intellectual disability and externalizing behavior problems started to become evident after 2 years of age. She presented her first seizures when she was 12-year-old, currently treated with levetiracetam (daily dose of 1000 mg *per os*, divided in twice), while aripiprazole (10 mg of the oral solution, once in a day) is at present administered to control her externalizing behavior disorder. ECG and echocardiogram were normal, except for mild valvular (both mitral and tricuspid) regurgitations. Brain MRI did not detect any abnormalities. a-CGH analysis evidenced no rearrangements, conversely, NGS analysis of a panel of genes involved in neurodevelopmental disorders identified a heterozygous variant of the *ANKRD11* gene (c.1902_1907del) (Ref Seq NM_013275, based on genome build GRCh37/hg19; rsID 886041125; ClinVar: RCV000011454.5). Such mutation is responsible for a frameshift, leading to the introduction of a premature stop codon at position 358 (p.Lys635fs) of the encoded protein. Also, a heterozygous missense mutation of the euchromatic histone lysine methyltransferase 1 (*EHMT1)* gene (c.103G > A) (p.Asp35Asn) (Ref Seq NM_024757, based on genome build GRCh37/hg19; rsID 371134699; ClinVar: RCV000011454.5), was found. Involvement of the *EHMT* gene, harboring on a highly conserved residue of the protein and with low frequency in the general population, has been associated with Kleefstra Syndrome (MIM #610253). The Platform used was Ion AmpliSeqCustom Panel version 4.44 - Torrent Personal Genome Machine (PGM) System, Ion Torrent Suite Software version 5; Genome Reference: hg19. Both coding regions and exon/intron junctions have been analyzed. Median coverage was 98%, with genes coverage between 84 and 99%, while the sequence coverage of single fragments analyzed ≥ 100X. NGS analysis of the same panel of genes has been performed also in the sister and showed normal results. Currently, the girl has concluded a high professional school with a dedicated support teacher, manifesting an externalizing behavior disorder especially related to interpersonal relationship issues characterized by physical aggressiveness, mainly with peers. She has a moderate degree of intellectual disability; she can carry out daily activities that allow personal autonomy, and complete manual tasks (manipulate and build simple objects, but not repair or creation); she is able as well of easy mathematic calculations and to use a personal computer for basic functions like writing, although with a short attention span.

### Patient 2

**Fig. 2a/b:**
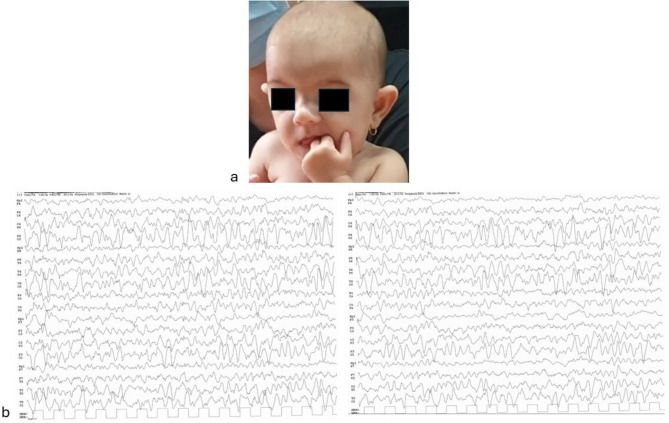
**a.** Patient 2 at 5 months of age. High forehead, prominent ears, wide nasal bridge and bulbous tip, thin upper lip. **b**. Moderately abnormal EEG pattern, due to bilateral posterior slow activity

The *proposita* is a three-year-old girl, fourth child of healthy non-consanguineous parents. She was born at 38 weeks of gestation, by caesarean delivery. Apgar scores were 8 and 9, at 1 and 5 min respectively. At birth, anthropometric measurements were as follows: weight 2490 g (7th centile, -1,44 SD), length 46,2 cm (10th centile, -1,29 SD), and OFC 35 cm (87th centile, 1,11 SD), according with the Italian Ines Growth Charts [9]. At our first observation at age 2 months and 12 days, she showed a growth delay: weight 3920 g (1st centile, -2,44 SD), length 53,5 cm (1st centile, -2,19 SD), and OFC 37 cm (8th centile, -1,39 SD), according to World Health Organization growth charts [8]. No cerebral anomalies were identified by head US. Neurodevelopmental evaluation showed a normal response to social smiling and vocalization, as well as a central hypotonia with axial distribution and tongue protrusion. No feeding difficulties were reported. She was enrolled in a neurodevelopmental follow-up. At 5 months and 9 days of age, her anthropometric measures were as follows: weight 5520 g (3rd centile, -1,96 SD), length 61 cm (6th centile, -1,58 SD) and OFC 41 cm (30th centile, -0,5 SD). On physical examination, high forehead with large anterior fontanelle, prominent ears, wide nasal bridge and bulbous tip along with thin upper lip were observed (Fig. 2a). Brachydactyly and bilateral clinodactyly of the fifth finger were also present.

Appropriate for the age neurodevelopmental milestones were acquired, although with persistence of a mild central axial hypotonia. Brainstem auditory evoked potentials were normal. At 13 months, an impairment of fine motor skills and reduced response readiness in interactions with the examiner were noted, with persistent mild central hypotonia and facial dysmorphic features along with delayed closure of the anterior fontanelle, suggesting further insights into the possible pathogenesis of her condition. An a-CGH analysis (100–150 Kb resolution, genomic assembly GRCh37.p13) identified a 16q24.3-24.3 deletion spanning 634 Kb, with the positions 88.873.958 and 89.507.835 being the breakpoints of the rearrangement. The deleted region involved several genes (*CDT1*,* APRT*,* GALNS*,* TRAPPC2L*,* ACSF3*,* CDH15*), besides *ANKRD11*, some of which have been associated with developmental disorders. The molecular karyotype performed in both parents did not reveal any genomic rearrangements. The patient was then enrolled in a psychomotor and neurocognitive stimulation program. Currently at 3 years of age, her anthropometric measures are as follows: weight 10,500 g (2nd centile, -2.15 SD), length 90 cm (12th centile, -1.17 SD) and OFC 49 cm (66th centile, + 0.4 SD). Her mother reports a moderate hyperactivity not complicated by oppositional behavior, along with bruxism and onychophagy. Neurodevelopmental evaluation was normal, with adequate interaction and participation during the developmental assessment. She does not present seizures, although the EEG pattern is moderately abnormal, for the presence of bilateral posterior slow activity (Fig. 2b).

She does not manifest any further clinical anomalies, and laboratory tests as well as US multiorgan evaluations (except for patent *foramen ovale* revealed by echocardiography) do not evidence other abnormalities to date.

### Patient 3

**Fig. 3a/b:**
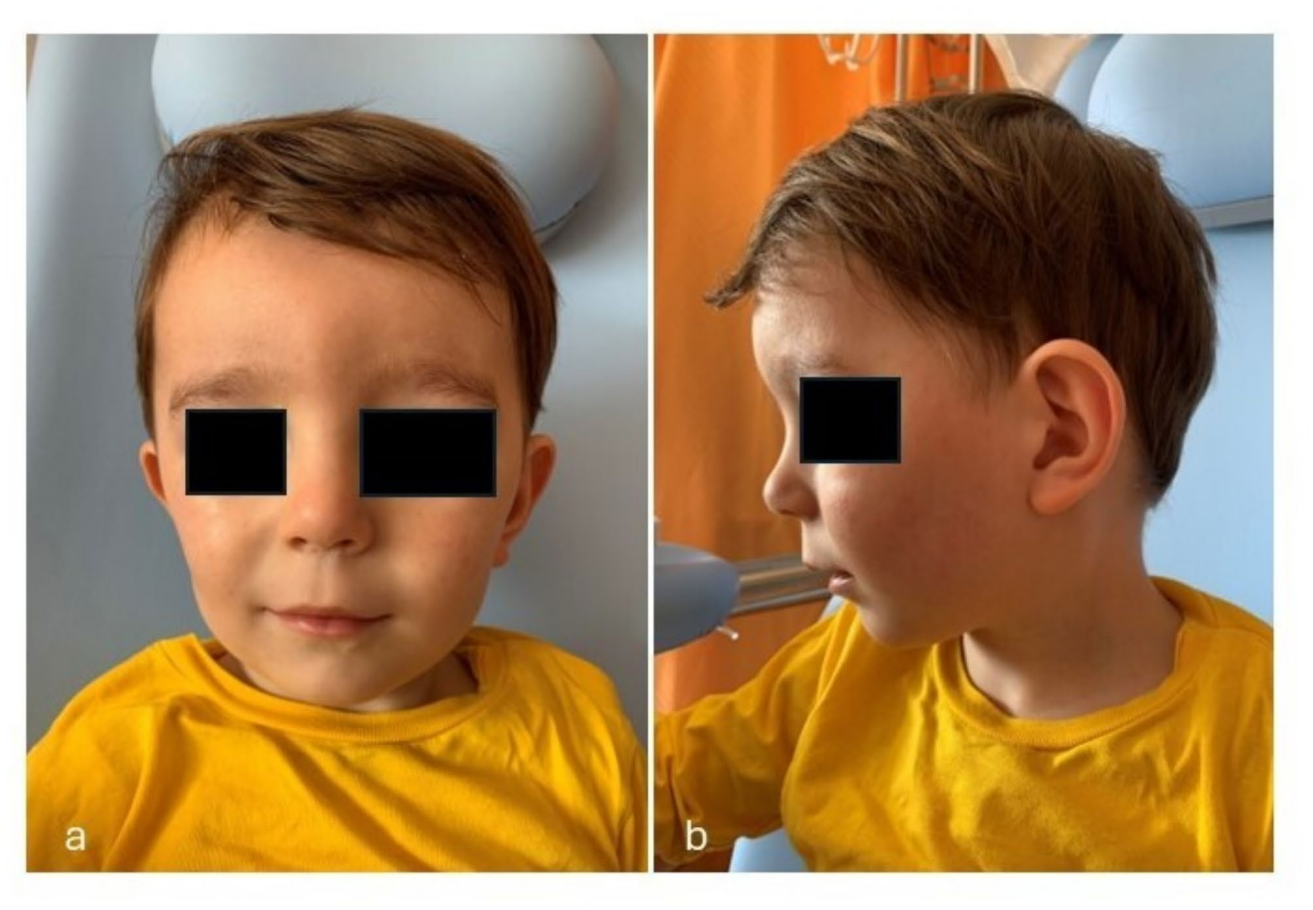
Patient 3, at 3 years and 6 months of age. **a** High forehead, squared face due to chubby cheeks and enlarged chin, thick and medially sparse eyebrows with barely detectable synophrys, wide nasal bridge with bulbous tip and anteverted nares, long philtrum, and thin upper lip. **b** prominent and wide ears with hypoplastic antihelix, and retrognathia

The proband is a 3-month-old male infant, second child of healthy non-consanguineous parents, both coming from Slovenia. Family history was unremarkable, including one healthy brother currently aged 8 years. He was born after a naturally conceived pregnancy, physiologically occurred until 38^+ 6^ weeks, when an emergency caesarean section was performed due to cardiotocographic abnormalities. For the increased risk of Down syndrome, revealed during the first trimester of pregnancy by the conventional multiple marker screening, genetic investigations through amniocentesis had been offered to the couple, and then performed: SNPs-array excluded genomic rearrangements (microdeletions and/or microduplications), and showed a normal male karyotype, 46 XY. Prenatal ultrasound evaluations, carried out in the following trimesters, documented polyhydramnios in addition to a previously evidenced fetal growth restriction (FGR), and raised the suspicion of rhizomelia of lower limbs. At birth, the newborn showed good adaptation to extrauterine life, with Apgar scores of 9 and 10 at 1 and 5 min, respectively. Anthropometric measures were as follows: weight 2324 g (1st centile, -2.51 SD), length 47.7 cm (9th centile, -1.34 SD), and OFC 33.8 cm (25th centile, -0.69 SD) [9]. He was soon transferred to the NICU due to dysmorphic features, and to continue the diagnostic work-up. At admission, he showed triangular face, large anterior fontanelle with wide metopic suture, and prominent ears. In addition, bifid tongue was observed. Bilateral brachydactyly along with clinodactyly of the 5th finger were also present, but no limbs abnormalities were identified. A scrotum anomaly, due to its superior margin upper to the base of the penis (shawl scrotum), was finally noted. Head and abdominal US evaluations documented no abnormalities, as well as total body X-ray (performed for the prenatal finding of rhizomelia) and ophthalmological assessment. Conversely, heart US evidenced a small membranous restrictive interventricular septal defect, while hearing screening through transient evoked otoacoustic emissions (TEOAE) revealed normal results. The patient was discharged from the NICU at 10 days of life and enrolled in a multidisciplinary follow-up. At 3 months of age an early teething was observed. Owing to such new findings, along with the already reported dysmorphic features, a genetic evaluation was performed, providing the indication to carry out Sanger sequencing of the *ANKRD11* gene. The genetic investigation identified the c.1903_1907delAAACA variant (p.Lys635GlnfsTer26) of the gene (Ref Seq NM_013275.6), for KBG syndrome diagnosis. Further genomic exams in the *trio* were refused by parents. Due to recurrent episodes of otitis, an audiological assessment through auditory brainstem response (ABR) evaluation was conducted at 3 years and 6 months of age. It detected a bilateral response threshold at 45 dB (decibel) HL (hearing level), according with mild conductive hypoacusis, which has not required any treatment to date. Clinical examination at that time disclosed additional dysmorphic features, including high forehead, squared face due to chubby cheeks and enlarged chin, prominent and wide ears with hypoplastic antihelix, thick and medially sparse eyebrows with barely detectable synophrys, wide nasal bridge with bulbous tip and anteverted nares. In addition, long philtrum, thin upper lip and retrognathia were also noted (Fig. 3a/b).

He is at present 4 years and 2 months old and shows normal growth - according to World Health Organization growth chart for neonatal and infant close monitoring [8]: weight 15 Kg (15th centile, -1.02 SD), height 101.7 cm (32nd centile, -0.46 SD) and OFC 51.5 cm (29th centile, -0.55 SD). On physical examination, macrodontia of the upper incisive teeth has been additionally observed. Furthermore, neurological assessment evidenced mild neuromotor delay, but neither seizures nor further anomalies are reported to date.

## Discussion and conclusions

KBG syndrome is characterized by distinctive craniofacial features including triangular face, synophrys, wide nasal bridge, macrodontia of upper central incisors, thin upper lip, in addition to short stature, developmental delay and intellectual disability. Seizures and EEG abnormalities may also be observed [10]. Skeletal defects like brachydactyly, clinodactyly, kyphosis, scoliosis and sternum abnormalities have also been reported [11]. KBG syndrome shows genetic heterogeneity along with phenotypical variability, the latter depending both on genomic alterations and time presentation. Diagnosis can be difficult, even more within the first months of life. In the present study we report on three unrelated patients showing different clinical pictures, in which diagnosis has been made at different developmental ages, from infancy to adolescence. Our patients manifested some distinctive facial features. Specifically, Patient 1 presented macrodontia, while our Patient 2 does not have to date, likely due to her young age, such highly suggestive clinical sign. In the literature, macrodontia is described as a typical sign of KBG syndrome, even if late. Other dentition disorders, including hyperdontia, oligodontia, as well as “shovel” shape, fusion and/or malposition of the incisors, have been observed [12, 13]. In our Patient 3, indeed, an early dentition was identified at 3 months of age. A large anterior fontanelle with delayed closure has been noted in Patients 2 and 3, and also this finding is reported in prior studies [10]. All our patients manifested brachydactyly and clinodactyly, according to literature data [10, 11, 14]. Each patient showed, as well, a neurodevelopmental involvement with different developmental trajectories and degree of severity related to the different age. Epilepsy and EEG abnormalities are widely described in previous reports [10, 11, 14]. Patient 1 experienced epileptic seizures, while the second proband showed EEG abnormalities. Some brain anomalies, such as hypoplasia of the cerebellar vermis, have been also well described [14]; in none of our patients, however, central nervous system (CNS) abnormalities have been found (Patient 1), or suggested by first-level imaging (head US) investigations. All three probands presented with cardiac anomalies, which conversely have been poorly recorded in literature reports to date [10]. Hearing loss following recurrent otitis has also been found [10, 11], as occurred in our Patient 3, in which a mild conductive hypoacusis has been identified at age 3 years. Finally, genital anomalies, and mainly cryptorchidism, are described in KBG syndrome patients [10, 11]. Our Patient 3 presented with shawl scrotum, which is rather characteristic of Aarskog syndrome [15]. The latter (in addition to other conditions like Cornelia de Lange, Coffin-Siris or Silver-Russell syndromes) shows many clinical features (short stature, facial dysmorphisms, macrodontia, brachydactyly, vertebral anomalies and cryptorchidism, but not intellectual disability) overlapping those of KBG syndrome, from which should be distinguished.

**Fig. 4:**
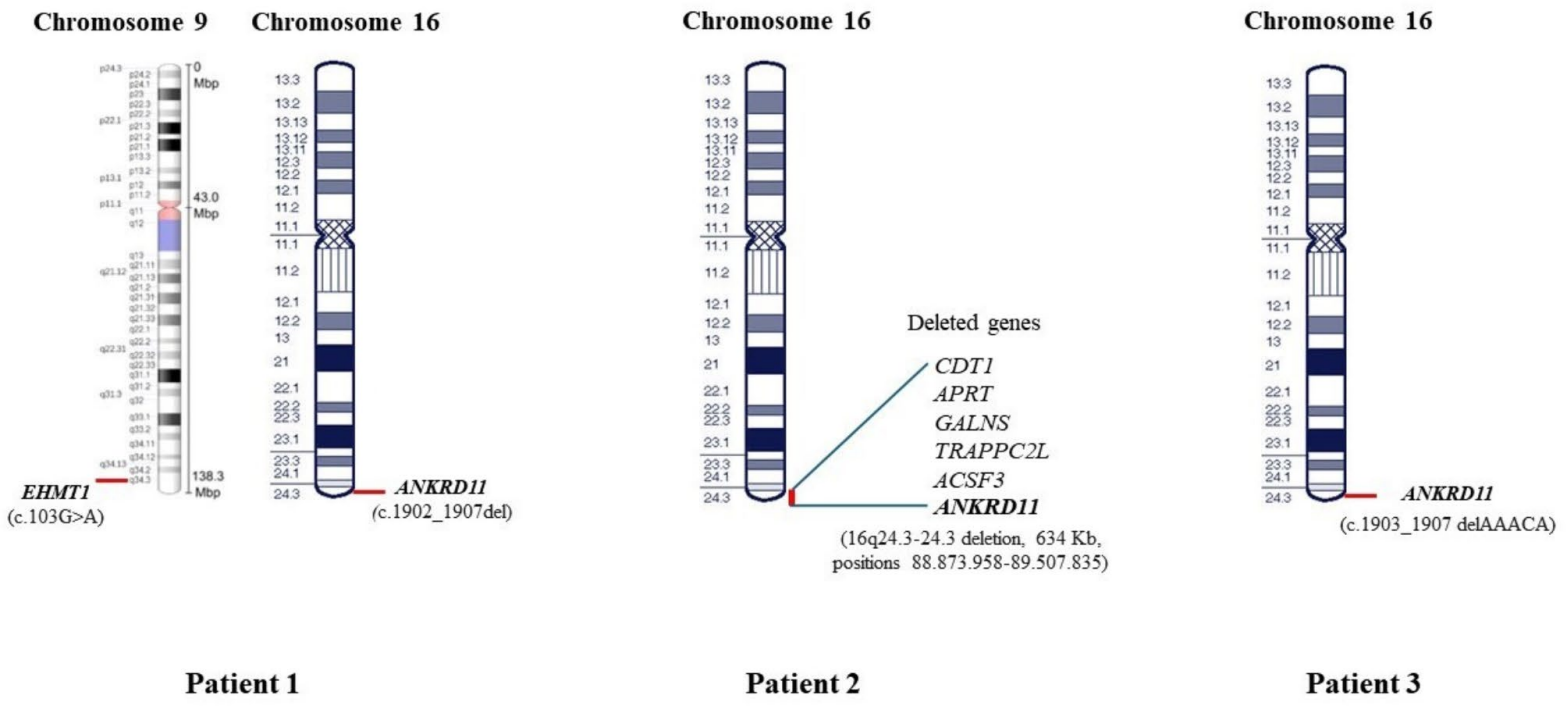
Map of the patients’pathogenic variants highlighting the genomic differences among them

Often the diagnosis is not made even long after the permanent teeth have erupted, as for Patient 1. However, it is crucial to pay attention to the dysmorphic phenotypical traits of the disease, which may raise less clearly in early infancy, as well as to its neuropsychological features, which by converse may more frequently appear over time. All three our patients, also on the basis of the recent review about KBG syndrome by Morel Swols et al. [10], presented with more than two suggestive clinical features, making the phenotypical picture compatible with such syndrome diagnosis. In particular, the variable association of macrodontia of upper incisors, developmental delay, postnatal short stature and peculiar facial dysmorphisms strengthened the pathogenic role of the genetic *ANKRD11* variants identified in the probands, along with the analysis of the dedicated genomic database confirming that such known mutations were disease-causing. A comprehensive and detailed comparison among our patients, including dysmorphic features, congenital defects, neuropsychological disorders, and genomic abnormalities (with the different genetic tests used for their detection), is reported and synthetized in Table 1; Fig. 4.

**Table 1:** Comparison among our three KBG patients

	Patient 1	Patient 2	Patient 3
**Craniofacial dysmorphic features**	• Squared face • Curly hair • Wide and prominent ears with hypoplastic antihelix • Thick eyebrows • Synophrys • Hypertelorism • Mild epicanthus • Wide nasal bridge and bulbous tip • Chubby cheeks • Hypoplastic and short philtrum • Macrodontia of permanent upper central incisors with dental crowding • Enlarged chin	• Brachycephaly • High forehead • Large anterior fontanelle with delayed closure • Wide and prominent ears • Bulbous nose • Thin upper lip	• Triangular face • Large anterior fontanelle with wide metopic suture • Prominent ears • Early teething • Bifid tongue
**Skeletal defects**	• Brachydactyly, clinodactyly of the 5th finger.	• Brachydactyly, clinodactyly of the 5th finger.	• Brachydactyly, clinodactyly of the 5th finger
**Cardiac abnormalities**	Mild valvular (both mitral and tricuspid) regurgitations	Patent *foramen ovale*	Small membranous restrictive interventricular septal defect
**Genital anomalies**	-	-	• Shawl scrotum
**Neuropsychological disorders**	• Neuromotor delay • Intellectual disability • Behavioral disorder • Seizures	• Generalized hypotonia • Mild developmental delay • EEG abnormalities	• Mild neuromotor delay
**Sensory defects**	-	-	Mild conductive hearing loss (following repeated otitis)
**Age at diagnosis**	18 years	13 months	3 months
**Genetic investigations**	NGS analysis of a panel of genes involved in neurodevelopmental disorders	a-CGH analysis	Sanger sequencing of *ANKRD11*
**Genes involved**	***ANKRD11****(*c.1902_1907del) ***EHMT1*** (c.103G > A)	**16q24.3-24.3 deletion** (634 Kb, within the positions 88.873.958 and 89.507.835, involving ***CDT1***,*** APRT***,*** GALNS***,*** TRAPPC2L***,*** ACSF3***,*** CDH15***, besides ***ANKRD11***)	***ANKRD11*** (c.1903_1907 delAAACA)

Besides the issues relating with the high phenotypical variability of the syndrome, moreover no clear genotype-phenotype correlations have been established to date, making thus difficult the diagnostic approach. Specifically, the syndrome has been associated with loss-of-function intragenic variant in *ANKRD11* [16]. Our Patient 3, indeed, whose complete genomic profile is however unknown, seems to have a “pure” clinical form caused by a single mutation in the *ANKRD11* gene, without additional clinical signs. Moreover, KBG syndrome has been linked with microdeletions or microduplications including the *ANKRD11* gene. Therefore, copy number variations (CNV) in the 16q24.3 region may be responsible for a variable phenotype, overlapping that of KBG syndrome. Its severity may be dependent on the genes included in the rearrangement, leading to complex (also to be recognized) clinical pictures according to contiguous gene syndromes [17–24]. Indeed, individuals with microdeletions show higher incidence of congenital heart defects, astigmatism, and thrombocytopenia than those with intragenic pathogenic variant. Furthermore, *CDH15* haploinsufficiency seems to contribute to a more severe neurological phenotype [25]. Therefore, we expect a disease progression in our second patient, who is carrier of a larger deletion including, besides *ANKRD11* and *CDH15*, other contiguous genes, and who is probably too young to completely express the phenotypical signs associated with the disease. Finally in Patient 1, the contextual presence of two mutations may have contributed to her more severe and complex phenotype, also if many of the clinical signs associated with mutations of the gene (*EHMT1*) responsible for Kleefstra syndrome (brachycephaly, unusual eyebrow shape, synophrys, cupid bow upper lip, full-everted lower lip, dental anomalies, hypotonia/motor delay, intellectual disability, speech disorder, congenital heart malformations, epilepsy, recurrent infections, hearing problems, and behavioral disturbances including aggressive/emotional outbursts in adolescence) are overlapping or similar to those of KBG. Such clinical overlap could make the phenomenon of dual diagnosis less evident in this case. Nonetheless, clinicians must consider this possibility, also in relation to the documented increase of these situations in the current era of next generation diagnostic technologies [26–35], which easily enable the recognition of multiple genetic diagnoses, including those that may have been unexpected, like occurred in our case. Conversely, it has been described a mild phenotype in patients carrying mosaicism for *ANKRD11* mutations, confirming that KBG clinical pictures might be dose dependent [36].

The diagnosis confirmation of KBG syndrome can be obtained by different molecular tests [37]; in our cases it was reached through Next Generation Sequencing (NGS), array comparative genomic hybridization (a-CGH) and Sanger sequencing of the *ANKRD11* gene. In the third case, the identification of highly suggestive features as early teething (a “handle” sign), together with the typical facial dysmorphisms recognized in early infancy, allowed a prompt diagnosis. Once the diagnosis is made, an accurate follow-up is essential, and should provide timely and long-term multidisciplinary evaluations, including neurological, auxological, orthopedic, odonto-stomatological, cardiological, otorhinolaryngological, and endocrinological assessments. Regarding the latter aspect, some KBG children affected also with short stature and treated with growth hormone therapy have been described. These studies seem to have demonstrated good results in term of prepubertal growth, with height after treatment (different groups with 3 or 5-year follow-up evaluations) close to the target height [38]. Such data suggest that *ANRKD11* mutations do not appear to limit the response to growth hormone treatment. However, further research is needed to define the optimal hormonal therapeutical strategy.

We underline similarities and differences among our patients, for both specific genetic and clinical characteristics, and the variable diagnostic paths used to reach the diagnosis. Pediatricians must be aware of KBG syndrome, and should be able to raise its diagnostic suspicion in the presence of peculiar facial dysmorphic features, short stature and neurological abnormalities including developmental delay, intellectual disability and epilepsy. Prompt diagnosis may enable better growth and development profiles for patients, and allow to control or limit some of the most relevant associated neurodevelopmental disorders which can appear over time, increasing the quality of life of the whole family.

## Data Availability

The datasets used and analyzed during the current study are available from the corresponding author on reasonable request.

## Abbreviations

a-CGH: Array comparative genomic hybridization
CNS: Central nervous system
CNV: Copy number variations
NGS: Next generation sequencing
OFC: Occipitofrontal circumference
PFO: Patent foramen ovale
SD: Standard deviations
TEOAE: Otoacoustic emissions
US: Ultrasound
WES: Whole exome sequencing
